# Combined targeting of HEDGEHOG signaling and BRD4 as a novel therapeutic option against melanoma

**DOI:** 10.18632/oncotarget.28441

**Published:** 2023-05-26

**Authors:** Silvia Pietrobono, Barbara Stecca

**Keywords:** melanoma, hedgehog signaling, BRD4, SOX2, GLI1

The Hedgehog-GLI (HH/GLI) pathway is aberrantly activated in several types of cancer. Canonical HH/GLI pathway is triggered by binding of HH ligands to the twelve-pass transmembrane receptor Patched 1 (PTCH1), which retrieves its inhibition on the seven-pass transmembrane G protein-coupled receptor Smoothened (SMO), leading to the activation of the GLI transcription factors. Small molecules inhibitors targeting the essential pathway transducer SMO (e.g., vismodegib, sonidegib) have demonstrated therapeutic efficacy in HH-dependent tumors, such as basal cell carcinoma (BCC) and medulloblastoma (MB). However, the therapeutic efficacy of these SMO antagonists is limited by the development of acquired resistance and recurrence after drug withdrawal, and by additional oncogenic signals responsible for non-canonical activation of GLI transcription factors [1]. We subscribe to the idea that targeting non-canonical HH/GLI signaling will improve the response rate and durability of therapeutic effects exerted by SMO inhibition. Therefore, the identification of novel targetable regulators that function downstream of SMO, especially those acting at the transcriptional level, is of critical importance to effectively inhibit the HH pathway and prevent tumor relapse.

Pietrobono and colleagues [2] significantly contributed to this field by describing the role performed by the transcription factor SOX2 in driving non-canonical activation of GLI1. The authors showed that SOX2 directly binds GLI1 proximal promoter enhancing its transcription and demonstrated that melanoma patients co-expressing both SOX2 and GLI1 transcripts are characterized by a shorter overall survival. Also, they provided evidence that the BET bromodomain protein BRD4 functions in a transcriptional complex with SOX2 in inducing non-canonical activation of GLI1. Previous work identified BET proteins as epigenetic regulators of HH transcriptional output and established the rationale for the use of BET inhibitors in cancers with active HH pathway [3]. This evidence strengthens the relevance of the findings by Pietrobono et al., shedding light on the potential application of SMO inhibitors in concert with BRD4 inhibitors. As a proof, authors showed that combined targeting of canonical HH/GLI signaling using the SMO inhibitor (SMOi) MRT-92 [4] and of the SOX2-BRD4 complex using a potent Proteolysis Targeted Chimeras (PROTAC)-derived BRD4 degrader (called MZ1) synergizes in inducing DNA damage in melanoma cells *in vitro* and translates into a marked antitumor activity in orthotopic melanoma models. The finding that GLI1 expression is completely inhibited by the MZ1/SMOi combination and that its ectopic expression completely rescues the effects of this combinatorial strategy confirmed GLI1 as the main molecular target of the SOX2-BRD4 transcriptional complex (Figure 1A).

**Figure 1:**
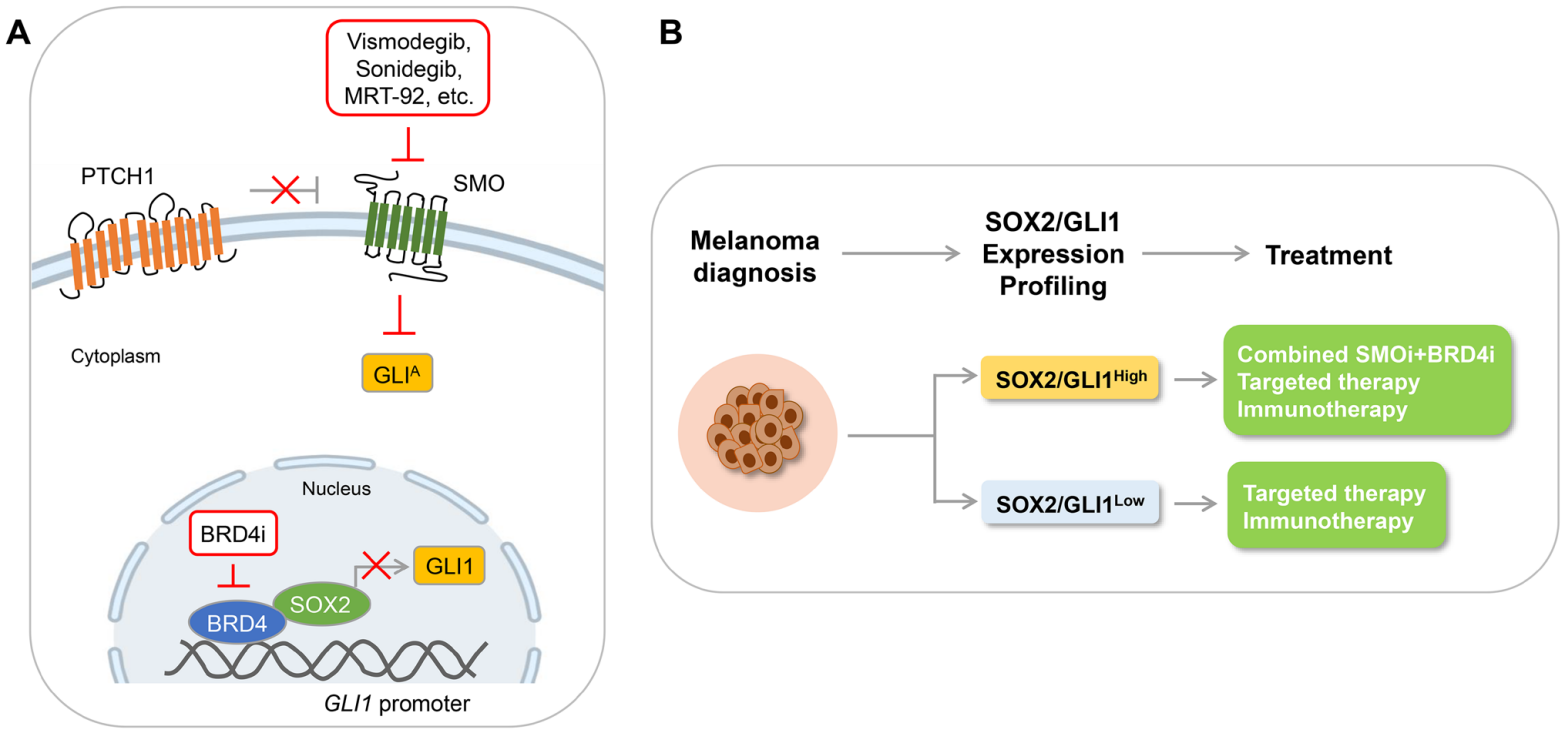
Combined targeting of SMO and BRD4 as a potential therapeutic strategy against melanoma. (**A**) Schematic diagram of the Hedgehog signaling, showing inhibition of the canonical pathway with SMO inhibitors and blockade of non-canonical activation of GLI1 with BRD4 inhibitors or PROTAC-based degraders. (**B**) Schematic diagram depicting therapeutic interventions in melanoma patients with high and low expression of SOX2/GLI1.

The findings by Pietrobono and colleagues are in line with a recent report showing that SHH subgroup MBs harbor a pool of SOX2+ cells in which the expression of GLI1 is increased by vismodegib treatment. Consistent with non-canonical SMO-independent activation of HH/GLI signaling, the proliferation of SOX2-enriched MB cells is not affected by SMO inhibition. Conversely, attenuation of GLI signaling using a clinically relevant BET inhibitor not only decreases vismodegib-sensitive and vismodegib-resistant primary MB growth but also reduces the number of SOX2+ cells and the chance of tumor recurrence [5].

Another important implication of the work by Pietrobono et al. is that the efficacy of MZ1/SMOi combinatorial treatment in melanoma is not influenced by *BRAF, NRAS* or *NF1* mutational status [2]. Despite advances in the treatment of metastatic melanoma, therapeutic options for patients with tumors that are BRAF and NRAS wild type remain limited [6]. This study opens the possibility of using this combination to treat melanoma expressing SOX2 and GLI1 irrespective to their mutational status.

Collectively, the findings presented by Pietrobono et al. pave the path for the development of a novel therapeutic strategy in tumors having both canonical and non-canonical HH/GLI signaling activation, such as melanoma. Indeed, only targeting non-canonical HH/GLI signaling could improve the response rates and durability of SMOi [2]. Because transcription factors like SOX2 are mostly undruggable due to the lack of active pockets or of ligand-binding domains, interfering with the SOX2-BRD4 axis represents a promising approach for treatment of refractory tumors addicted to the activity of this transcription factor (Figure 1B). While PROTAC-derived BRD4 degraders are in preclinical development [7, 8], several SMO inhibitors have already been approved for treatment of advanced BCC. Therefore, future studies are warranted to validate the combination of BRD4 and SMO inhibitors in melanoma patients and other cancer types with hyperactivation of the SOX2-BRD4-GLI1 axis.

## References

[1] Pietrobono S , et al. Front Genet. 2019; 10:556. 10.3389/fgene.2019.00556. 31244888 PMC6581679

[2] Pietrobono S , et al. Oncogene. 2021; 40:3799–814. 10.1038/s41388-021-01783-9. 33958721 PMC8175236

[3] Tang Y , et al. Nat Med. 2014; 20:732–40. 10.1038/nm.3613. 24973920 PMC4108909

[4] Pietrobono S , et al. Cell Death Dis. 2018; 9:142. 10.1038/s41419-017-0142-0. 29396391 PMC5833413

[5] Swiderska-Syn M , et al. Sci Adv. 2022; 8:eabj9138. 10.1126/sciadv.abj9138. 35857834 PMC9299538

[6] Dummer R , et al. Ann Oncol. 2012 (Suppl 7); 23:vii86–91. 10.1093/annonc/mds229. 22997461

[7] Liu Z , et al. Mol Biomed. 2022; 3:46. 10.1186/s43556-022-00112-0. 36536188 PMC9763089

[8] Schwalm MP , et al. Curr Opin Chem Biol. 2022; 68:102148. 10.1016/j.cbpa.2022.102148. 35462054

